# *rac*-*syn*-Diethyl 2-hy­droxy-4-oxo-1-phenyl­cyclo­hexane-1,2-dicarboxyl­ate

**DOI:** 10.1107/S1600536811042048

**Published:** 2011-10-22

**Authors:** Ya-Jun Wang, Sheng-Liang Ni, Yue Meng

**Affiliations:** a Department of Chemistry, Huzhou University, Huzhou, Zhejiang 313000, People’s Republic of China

## Abstract

The title compound, C_18_H_22_O_6_, was obtained by the domino oxa–Michael–aldol (DOMA) reaction and has the cyclo­hexa­none ring in a chair conformation with intra-annular torsion angles in the range 49.9 (2)–58.9 (2)°. The two eth­oxy­carbonyl substituents on the cyclo­hexa­none ring adopt a *syn* configurations. In the crystal, the mol­ecules self-assemble through duplex inter­molecular hy­droxy–carbonyl O—H⋯O hydrogen bonds, giving centrosymmetric cyclic dimers [graph set *R*_2_^2^(12)] which inter-associate through weak C—H⋯O hydrogen-bonding inter­actions.

## Related literature

For general background to proline-catalysed Robinson annulation, see: Eder *et al.* (1971 ▶); Hajos & Parrish (1974 ▶). For the catalytic asymmetric formation of chiral building blocks, see: Bui & Barbas (2000 ▶); Tanaka *et al.* (2003 ▶). For the the DOMA reaction, see: Nising & Bräse (2008) ▶; Sefer *et al.* (2010 ▶) and for asymmetric C—C bond-forming reactions, see: Sibi & Chen (2001 ▶); Tian *et al.* (2002 ▶); Gothelf *et al.* (2002 ▶); Rueping *et al.* (2009 ▶). For the synthesis of the title compound, see: Floyd & Miller (1963 ▶). For related structures, see: Abell *et al.* (1988 ▶); Hernández-Ortega *et al.* (2001 ▶). For graph-set analysis, see: Etter *et al.* (1990 ▶).
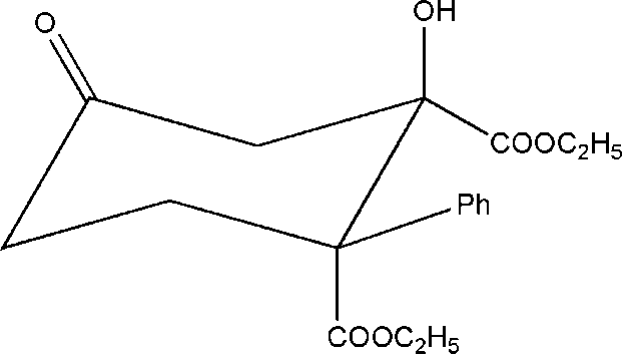


## Experimental

### 

#### Crystal data

C_18_H_22_O_6_
*M* *_r_* = 334.36
Triclinic, 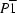

*a* = 8.2069 (10) Å
*b* = 9.9393 (16) Å
*c* = 11.1420 (17) Å
α = 87.408 (10)°
β = 70.610 (7)°
γ = 78.983 (9)°
*V* = 841.3 (2) Å^3^
*Z* = 2
Mo *K*α radiation
μ = 0.10 mm^−1^
*T* = 153 K
0.45 × 0.36 × 0.10 mm

#### Data collection

Rigaku R-AXIS RAPID CCD diffractometer
Absorption correction: multi-scan (*ABSCOR*; Higashi, 1995 ▶) *T*_min_ = 0.957, *T*_max_ = 0.990
8251 measured reflections
3053 independent reflections
2695 reflections with *I* > 2σ(*I*)
*R*_int_ = 0.019

#### Refinement

*R*[*F* ^2^ > 2σ(*F* ^2^)] = 0.042
*wR*(*F* ^2^) = 0.103
*S* = 1.07
3053 reflections
221 parameters
H-atom parameters constrained
Δρ_max_ = 0.46 e Å^−3^
Δρ_min_ = −0.29 e Å^−3^

### 

Data collection: *RAPID-AUTO* (Rigaku, 1998 ▶); cell refinement: *RAPID-AUTO*; data reduction: *CrystalStructure* (Rigaku/MSC, 2004 ▶); program(s) used to solve structure: *SHELXS97* (Sheldrick, 2008 ▶); program(s) used to refine structure: *SHELXL97* (Sheldrick, 2008 ▶); molecular graphics: *SHELXTL* (Sheldrick, 2008 ▶); software used to prepare material for publication: *SHELXL97*.

## Supplementary Material

Crystal structure: contains datablock(s) global, I. DOI: 10.1107/S1600536811042048/zs2151sup1.cif

Structure factors: contains datablock(s) I. DOI: 10.1107/S1600536811042048/zs2151Isup2.hkl

Supplementary material file. DOI: 10.1107/S1600536811042048/zs2151Isup3.cml

Additional supplementary materials:  crystallographic information; 3D view; checkCIF report

## Figures and Tables

**Table 1:** Hydrogen-bond geometry (Å, °)

*D*—H⋯*A*	*D*—H	H⋯*A*	*D*⋯*A*	*D*—H⋯*A*
O2—H2⋯O1^i^	0.84	1.91	2.7491 (18)	177
C15—H15⋯O2^ii^	0.95	2.55	3.486 (2)	169
C8—H8*A*⋯O5^iii^	0.99	2.44	3.095 (2)	124

Symmetry codes: (i) 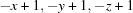; (ii) 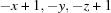; (iii) 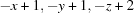.

**Fig. 1.**
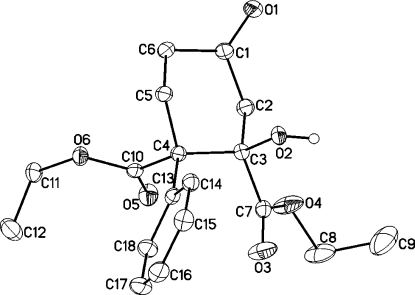
ORTEP view of the title compound. Non-associative hydrogen atoms are omitted and displacement ellipsoids are drawn at the 45% probability level.

**Fig. 2.**
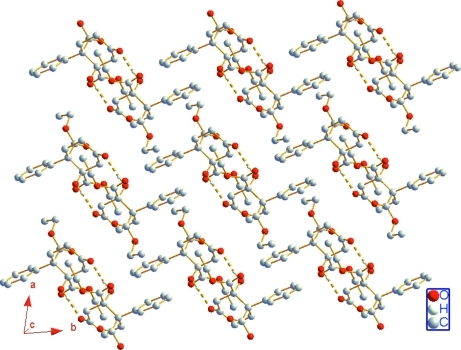
The hydrogen-bonding associations in the title compound with the O—H···O hydrogen bonds indicated by dashed lines.

## supplementary crystallographic information

### Comment

The domino oxa-Michael-aldol (DOMA) reaction reported herein is related to the proline–catalyzed Robinson annulation pioneered by Wiechert and by Hajos 40 years ago (Eder *et al.*, 1971, Hajos *et al.*, 1974). The catalytic asymmetric formation of chiral building blocks (Bui & Barbas, 2000; Tanaka *et al.*, 2003) represents an increasingly important field in pharmaceutical and organic chemistry owing to the usefulness of these products in further synthetic transformations. To date, the DOMA reaction has attracted considerable attention since they allow an efficient access to highly functionalized scaffolds, which occur in a variety of natural compounds with high biological activities (Nising & Bräse, 2008; Sefer *et al.*, 2010). Among the various asymmetric C—C bond-forming reactions, the direct catalytic domino (Sibi & Chen, 2001; Tian *et al.*, 2002) and cycloaddition reactions (Gothelf *et al.*, 2002; Rueping *et al.*, 2009) are of particular interest since multiple stereogenic centers can be formed in a single reaction.

Here we report the synthesis and structure of the title compound C_18_H_22_O_6_, racemic (*syn*)-3,4-diethoxycarbonyl-3-hydroxy-4-phenylcyclohexanone, a novel compound which has an interesting application in the synthesis of substituted hydrophenanthrene derivatives. Fortunately, the intramolecular aldol reaction proceeds in a highly diastereoselective fashion to form the six-membered ring so that all large substituents are equatorial and thus are controlled by the stable stereogenic center formed in the initial Michael reaction. The crystal structure of the title compound is reported here.

The asymmetric unit this compound consists of a molecule having the *syn* configuration (Fig. 1). The cyclohexanone ring adopts a chair conformation with intra-annular torsion angles in the range 49.9 (2)– 58.9 (2) ° [mean 53.9 (2)°], which have the expected values (Abell *et al.*, 1988). The attached 3-hydroxy and 4-phenyl groups are disposed in α-axial and β-equatorial configurations, respectively, while the two ethoxycarbonyl groups adopt *syn* configurations (Hernández-Ortega *et al.*, 2001). In the crystal, two molecules are connected through duplex intermolecular hydroxyl–carbonyl O—H···O hydrogen bonds (Table 1) giving centrosymmetric cyclic dimers [graph set *R*^2^_2_(12) (Etter *et al.*, 1990)] (Fig. 2), which inter-associate through weak C—H···O hydrogen-bonding interactions.

### Experimental

Diethyl 2-oxo-3-phenylsuccinate is prepared by condensation of ethyl 2–phenylacetate and diethyl oxalate (Floyd & Miller, 1963). To a suspension of proline (0.069 g, 0.6 mmol) in methyl vinyl α–ketone (0.28 g, 4 mmol) was added diethyl 2-oxo-3-phenylsuccinate (0.53 g, 2 mmol). The resulting mixture was stirred for five hours in a closed vessel with the reaction progress monitored by TLC. After the acceptor ketoester was consumed, the reaction mixture was treated with saturated ammonium chloride solution, then the organic layer was separated, and the aqueous layer was extracted with ethyl acetate three times. The combined organic layers were dried over anhydrous sodium sulfate. After removal of solvent, the residue was purified using column chromatography on silica gel (eluent: ethyl acetate/petroleum ether = 1/5, V/V) to give the light-yellow *syn*-adduct (I) in 51.4% yield and the *anti*-adduct (II) in 4.47% yield. The colorless needle-like crystals of (I) were obtained by slowly evaporating a solution of in the mixed solvent (ethyl acetate/petroleum ether = 1/5, V/V) at room temperature for two weeks.

### Refinement

All H-atoms bonded to C were positioned geometrically and refined using a riding model with d(C—H) = 0.93–0.99 Å and *U*_iso_(H) = 1.2*U*_eq_(C) (aromatic and methine) or 1.5*U*_eq_(C) (methyl). The hydroxy H was located in a difference Fourier synthesis and was refined using a riding model, with O—H fixed as initially found and with *U*_iso_(H) = 1.2*U*_eq_(O).

### Crystal data

**Table:**

C_18_H_22_O_6_	*Z* = 2
*M**_r_* = 334.36	*F*(000) = 356
Triclinic, *P*1	*D*_x_ = 1.320 Mg m^−^^3^
Hall symbol: -P 1	Mo *K*α radiation, λ = 0.71070 Å
*a* = 8.2069 (10) Å	Cell parameters from 3233 reflections
*b* = 9.9393 (16) Å	θ = 3.1–25.3°
*c* = 11.1420 (17) Å	µ = 0.10 mm^−^^1^
α = 87.408 (10)°	*T* = 153 K
β = 70.610 (7)°	Needle-like, colorless
γ = 78.983 (9)°	0.45 × 0.36 × 0.10 mm
*V* = 841.3 (2) Å^3^	

### Data collection

**Table:**

Rigaku R-AXIS RAPID CCD diffractometer	3053 independent reflections
Radiation source: fine-focus sealed tube	2695 reflections with *I* > 2σ(*I*)
graphite	*R*_int_ = 0.019
Detector resolution: 7.31 pixels mm^-1^	θ_max_ = 25.4°, θ_min_ = 3.2°
ω scans	*h* = −9→9
Absorption correction: multi-scan (*ABSCOR*; Higashi, 1995)	*k* = −11→11
*T*_min_ = 0.957, *T*_max_ = 0.990	*l* = −13→13
8251 measured reflections	

### Refinement

**Table:**

Refinement on *F*^2^	Primary atom site location: structure-invariant direct methods
Least-squares matrix: full	Secondary atom site location: difference Fourier map
*R*[*F*^2^ > 2σ(*F*^2^)] = 0.042	Hydrogen site location: inferred from neighbouring sites
*wR*(*F*^2^) = 0.103	H-atom parameters constrained
*S* = 1.07	*w* = 1/[σ^2^(*F*_o_^2^) + (0.0431*P*)^2^ + 0.4508*P*] where *P* = (*F*_o_^2^ + 2*F*_c_^2^)/3
3053 reflections	(Δ/σ)_max_ < 0.001
221 parameters	Δρ_max_ = 0.46 e Å^−^^3^
0 restraints	Δρ_min_ = −0.29 e Å^−^^3^

### Special details

**Table:**

Geometry. All e.s.d.'s (except the e.s.d. in the dihedral angle between two l.s. planes) are estimated using the full covariance matrix. The cell e.s.d.'s are taken into account individually in the estimation of e.s.d.'s in distances, angles and torsion angles; correlations between e.s.d.'s in cell parameters are only used when they are defined by crystal symmetry. An approximate (isotropic) treatment of cell e.s.d.'s is used for estimating e.s.d.'s involving l.s. planes.
Refinement. Refinement of *F*^2^ against ALL reflections. The weighted *R*-factor *wR* and goodness of fit *S* are based on *F*^2^, conventional *R*-factors *R* are based on *F*, with *F* set to zero for negative *F*^2^. The threshold expression of *F*^2^ > σ(*F*^2^) is used only for calculating *R*-factors(gt) *etc*. and is not relevant to the choice of reflections for refinement. *R*-factors based on *F*^2^ are statistically about twice as large as those based on *F*, and *R*- factors based on ALL data will be even larger.

### Fractional atomic coordinates and isotropic or equivalent isotropic displacement parameters (Å^2^)

**Table:**

	*x*	*y*	*z*	*U*_iso_*/*U*_eq_	
O1	0.79576 (15)	0.51980 (12)	0.38078 (11)	0.0255 (3)	
O2	0.51399 (14)	0.29561 (11)	0.57801 (10)	0.0192 (3)	
H2	0.4211	0.3544	0.5918	0.029*	
O3	0.41781 (18)	0.26892 (13)	0.85521 (12)	0.0369 (3)	
O4	0.45085 (18)	0.48608 (12)	0.82340 (12)	0.0374 (4)	
O5	0.83175 (15)	0.31071 (12)	0.79925 (11)	0.0261 (3)	
O6	1.03868 (14)	0.14904 (11)	0.67206 (10)	0.0207 (3)	
C1	0.8099 (2)	0.43872 (16)	0.46397 (15)	0.0186 (3)	
C2	0.6860 (2)	0.45938 (15)	0.59878 (15)	0.0194 (3)	
H2A	0.5868	0.5351	0.6018	0.023*	
H2B	0.7486	0.4865	0.6533	0.023*	
C3	0.61292 (19)	0.32920 (15)	0.65220 (14)	0.0164 (3)	
C4	0.76503 (19)	0.20183 (15)	0.63581 (14)	0.0158 (3)	
C5	0.8748 (2)	0.18687 (15)	0.49233 (14)	0.0180 (3)	
H5A	0.9718	0.1068	0.4794	0.022*	
H5B	0.7992	0.1689	0.4440	0.022*	
C6	0.9526 (2)	0.31423 (16)	0.43916 (15)	0.0206 (3)	
H6A	1.0393	0.3268	0.4798	0.025*	
H6B	1.0143	0.3016	0.3464	0.025*	
C7	0.4867 (2)	0.35472 (16)	0.79048 (15)	0.0183 (3)	
C8	0.3101 (3)	0.5304 (2)	0.94311 (18)	0.0439 (5)	
H8A	0.3523	0.5849	0.9949	0.053*	
H8B	0.2737	0.4495	0.9923	0.053*	
C9	0.1584 (3)	0.6149 (3)	0.9138 (2)	0.0568 (7)	
H9A	0.1967	0.6922	0.8614	0.085*	
H9B	0.0659	0.6496	0.9932	0.085*	
H9C	0.1124	0.5586	0.8673	0.085*	
C10	0.8800 (2)	0.22881 (15)	0.71235 (14)	0.0168 (3)	
C11	1.1587 (2)	0.15987 (18)	0.74163 (16)	0.0234 (4)	
H11A	1.1362	0.2549	0.7744	0.028*	
H11B	1.2816	0.1383	0.6834	0.028*	
C12	1.1337 (3)	0.0626 (2)	0.85057 (19)	0.0361 (5)	
H12A	1.0160	0.0902	0.9128	0.054*	
H12B	1.2223	0.0646	0.8913	0.054*	
H12C	1.1465	−0.0305	0.8188	0.054*	
C13	0.69874 (19)	0.06732 (15)	0.68411 (14)	0.0169 (3)	
C14	0.6427 (2)	−0.00903 (16)	0.60868 (15)	0.0198 (3)	
H14	0.6423	0.0232	0.5272	0.024*	
C15	0.5873 (2)	−0.13174 (16)	0.65096 (17)	0.0246 (4)	
H15	0.5487	−0.1821	0.5985	0.030*	
C16	0.5881 (2)	−0.18106 (17)	0.76895 (17)	0.0265 (4)	
H16	0.5515	−0.2655	0.7972	0.032*	
C17	0.6423 (2)	−0.10641 (18)	0.84515 (16)	0.0272 (4)	
H17	0.6421	−0.1391	0.9267	0.033*	
C18	0.6974 (2)	0.01642 (17)	0.80311 (15)	0.0224 (4)	
H18	0.7348	0.0666	0.8564	0.027*	

### Atomic displacement parameters (Å^2^)

**Table:**

	*U*^11^	*U*^22^	*U*^33^	*U*^12^	*U*^13^	*U*^23^
O1	0.0242 (6)	0.0241 (6)	0.0227 (6)	0.0003 (5)	−0.0040 (5)	0.0062 (5)
O2	0.0176 (6)	0.0195 (6)	0.0212 (6)	0.0007 (4)	−0.0093 (5)	−0.0015 (4)
O3	0.0416 (8)	0.0258 (7)	0.0282 (7)	−0.0073 (6)	0.0087 (6)	0.0009 (5)
O4	0.0479 (8)	0.0205 (6)	0.0248 (7)	−0.0024 (6)	0.0120 (6)	−0.0062 (5)
O5	0.0252 (6)	0.0276 (6)	0.0254 (6)	0.0010 (5)	−0.0105 (5)	−0.0083 (5)
O6	0.0163 (6)	0.0231 (6)	0.0228 (6)	−0.0004 (4)	−0.0081 (5)	−0.0006 (5)
C1	0.0192 (8)	0.0168 (8)	0.0207 (8)	−0.0063 (6)	−0.0063 (7)	0.0025 (6)
C2	0.0197 (8)	0.0146 (7)	0.0210 (8)	−0.0006 (6)	−0.0044 (7)	0.0002 (6)
C3	0.0163 (7)	0.0161 (8)	0.0172 (8)	−0.0021 (6)	−0.0066 (6)	0.0001 (6)
C4	0.0159 (7)	0.0142 (7)	0.0162 (7)	−0.0009 (6)	−0.0047 (6)	−0.0005 (6)
C5	0.0189 (8)	0.0159 (8)	0.0164 (8)	−0.0003 (6)	−0.0035 (6)	−0.0003 (6)
C6	0.0172 (8)	0.0212 (8)	0.0195 (8)	−0.0014 (6)	−0.0024 (6)	0.0025 (6)
C7	0.0169 (8)	0.0177 (8)	0.0197 (8)	−0.0004 (6)	−0.0068 (6)	−0.0011 (6)
C8	0.0530 (13)	0.0310 (10)	0.0244 (10)	0.0039 (9)	0.0126 (9)	−0.0065 (8)
C9	0.0353 (12)	0.0859 (19)	0.0386 (12)	−0.0001 (12)	−0.0013 (10)	−0.0235 (12)
C10	0.0161 (8)	0.0159 (7)	0.0168 (8)	−0.0034 (6)	−0.0034 (6)	0.0037 (6)
C11	0.0173 (8)	0.0287 (9)	0.0272 (9)	−0.0050 (7)	−0.0113 (7)	0.0031 (7)
C12	0.0361 (11)	0.0402 (11)	0.0400 (11)	−0.0121 (9)	−0.0223 (9)	0.0165 (9)
C13	0.0135 (7)	0.0154 (7)	0.0196 (8)	−0.0007 (6)	−0.0034 (6)	−0.0007 (6)
C14	0.0187 (8)	0.0189 (8)	0.0208 (8)	−0.0013 (6)	−0.0065 (7)	−0.0004 (6)
C15	0.0237 (9)	0.0191 (8)	0.0318 (9)	−0.0042 (7)	−0.0097 (7)	−0.0033 (7)
C16	0.0236 (9)	0.0181 (8)	0.0354 (10)	−0.0071 (7)	−0.0053 (7)	0.0040 (7)
C17	0.0306 (9)	0.0263 (9)	0.0239 (9)	−0.0076 (7)	−0.0076 (7)	0.0071 (7)
C18	0.0248 (9)	0.0212 (8)	0.0224 (8)	−0.0060 (7)	−0.0086 (7)	0.0021 (7)

### Geometric parameters (Å, °)

**Table:**

O1—C1	1.2206 (19)	C8—C9	1.486 (3)
O2—C3	1.4241 (18)	C8—H8A	0.9900
O2—H2	0.8400	C8—H8B	0.9900
O3—C7	1.194 (2)	C9—H9A	0.9800
O4—C7	1.3230 (19)	C9—H9B	0.9800
O4—C8	1.464 (2)	C9—H9C	0.9800
O5—C10	1.2043 (19)	C11—C12	1.501 (2)
O6—C10	1.3326 (19)	C11—H11A	0.9900
O6—C11	1.4617 (19)	C11—H11B	0.9900
C1—C6	1.497 (2)	C12—H12A	0.9800
C1—C2	1.505 (2)	C12—H12B	0.9800
C2—C3	1.542 (2)	C12—H12C	0.9800
C2—H2A	0.9900	C13—C14	1.393 (2)
C2—H2B	0.9900	C13—C18	1.395 (2)
C3—C7	1.545 (2)	C14—C15	1.391 (2)
C3—C4	1.568 (2)	C14—H14	0.9500
C4—C10	1.530 (2)	C15—C16	1.384 (2)
C4—C13	1.546 (2)	C15—H15	0.9500
C4—C5	1.550 (2)	C16—C17	1.380 (3)
C5—C6	1.533 (2)	C16—H16	0.9500
C5—H5A	0.9900	C17—C18	1.390 (2)
C5—H5B	0.9900	C17—H17	0.9500
C6—H6A	0.9900	C18—H18	0.9500
C6—H6B	0.9900		
			
C3—O2—H2	109.5	O4—C8—H8B	109.9
C7—O4—C8	117.92 (14)	C9—C8—H8B	109.9
C10—O6—C11	117.03 (12)	H8A—C8—H8B	108.3
O1—C1—C6	122.31 (14)	C8—C9—H9A	109.5
O1—C1—C2	121.82 (14)	C8—C9—H9B	109.5
C6—C1—C2	115.86 (13)	H9A—C9—H9B	109.5
C1—C2—C3	112.37 (13)	C8—C9—H9C	109.5
C1—C2—H2A	109.1	H9A—C9—H9C	109.5
C3—C2—H2A	109.1	H9B—C9—H9C	109.5
C1—C2—H2B	109.1	O5—C10—O6	124.40 (14)
C3—C2—H2B	109.1	O5—C10—C4	124.04 (14)
H2A—C2—H2B	107.9	O6—C10—C4	111.56 (13)
O2—C3—C2	108.84 (12)	O6—C11—C12	110.41 (14)
O2—C3—C7	107.31 (12)	O6—C11—H11A	109.6
C2—C3—C7	110.70 (12)	C12—C11—H11A	109.6
O2—C3—C4	104.69 (11)	O6—C11—H11B	109.6
C2—C3—C4	111.26 (12)	C12—C11—H11B	109.6
C7—C3—C4	113.68 (12)	H11A—C11—H11B	108.1
C10—C4—C13	107.52 (12)	C11—C12—H12A	109.5
C10—C4—C5	109.82 (12)	C11—C12—H12B	109.5
C13—C4—C5	110.10 (12)	H12A—C12—H12B	109.5
C10—C4—C3	108.55 (12)	C11—C12—H12C	109.5
C13—C4—C3	113.42 (12)	H12A—C12—H12C	109.5
C5—C4—C3	107.40 (12)	H12B—C12—H12C	109.5
C6—C5—C4	113.04 (12)	C14—C13—C18	117.75 (14)
C6—C5—H5A	109.0	C14—C13—C4	121.03 (14)
C4—C5—H5A	109.0	C18—C13—C4	121.20 (14)
C6—C5—H5B	109.0	C15—C14—C13	120.96 (15)
C4—C5—H5B	109.0	C15—C14—H14	119.5
H5A—C5—H5B	107.8	C13—C14—H14	119.5
C1—C6—C5	110.24 (12)	C16—C15—C14	120.46 (16)
C1—C6—H6A	109.6	C16—C15—H15	119.8
C5—C6—H6A	109.6	C14—C15—H15	119.8
C1—C6—H6B	109.6	C17—C16—C15	119.34 (16)
C5—C6—H6B	109.6	C17—C16—H16	120.3
H6A—C6—H6B	108.1	C15—C16—H16	120.3
O3—C7—O4	124.13 (15)	C16—C17—C18	120.26 (16)
O3—C7—C3	124.09 (14)	C16—C17—H17	119.9
O4—C7—C3	111.44 (13)	C18—C17—H17	119.9
O4—C8—C9	108.77 (17)	C17—C18—C13	121.22 (15)
O4—C8—H8A	109.9	C17—C18—H18	119.4
C9—C8—H8A	109.9	C13—C18—H18	119.4
			
O1—C1—C2—C3	131.17 (15)	C2—C3—C7—O4	9.43 (18)
C6—C1—C2—C3	−49.84 (18)	C4—C3—C7—O4	135.52 (14)
C1—C2—C3—O2	−62.33 (16)	C7—O4—C8—C9	−110.0 (2)
C1—C2—C3—C7	179.96 (13)	C11—O6—C10—O5	2.0 (2)
C1—C2—C3—C4	52.53 (17)	C11—O6—C10—C4	−177.03 (12)
O2—C3—C4—C10	179.76 (11)	C13—C4—C10—O5	−101.99 (17)
C2—C3—C4—C10	62.36 (16)	C5—C4—C10—O5	138.22 (15)
C7—C3—C4—C10	−63.44 (16)	C3—C4—C10—O5	21.1 (2)
O2—C3—C4—C13	−60.81 (15)	C13—C4—C10—O6	77.06 (15)
C2—C3—C4—C13	−178.21 (12)	C5—C4—C10—O6	−42.73 (16)
C7—C3—C4—C13	55.99 (17)	C3—C4—C10—O6	−159.87 (12)
O2—C3—C4—C5	61.07 (14)	C10—O6—C11—C12	88.62 (17)
C2—C3—C4—C5	−56.33 (16)	C10—C4—C13—C14	−158.13 (14)
C7—C3—C4—C5	177.88 (12)	C5—C4—C13—C14	−38.52 (19)
C10—C4—C5—C6	−59.02 (16)	C3—C4—C13—C14	81.85 (17)
C13—C4—C5—C6	−177.22 (12)	C10—C4—C13—C18	20.14 (19)
C3—C4—C5—C6	58.84 (16)	C5—C4—C13—C18	139.75 (15)
O1—C1—C6—C5	−130.89 (16)	C3—C4—C13—C18	−99.88 (17)
C2—C1—C6—C5	50.13 (18)	C18—C13—C14—C15	0.0 (2)
C4—C5—C6—C1	−55.46 (17)	C4—C13—C14—C15	178.36 (14)
C8—O4—C7—O3	−4.0 (3)	C13—C14—C15—C16	−0.5 (2)
C8—O4—C7—C3	169.59 (16)	C14—C15—C16—C17	0.8 (3)
O2—C3—C7—O3	64.36 (19)	C15—C16—C17—C18	−0.7 (3)
C2—C3—C7—O3	−176.99 (15)	C16—C17—C18—C13	0.2 (3)
C4—C3—C7—O3	−50.9 (2)	C14—C13—C18—C17	0.1 (2)
O2—C3—C7—O4	−109.22 (14)	C4—C13—C18—C17	−178.23 (15)

### Hydrogen-bond geometry (Å, °)

**Table:**

*D*—H···*A*	*D*—H	H···*A*	*D*···*A*	*D*—H···*A*
O2—H2···O1^i^	0.84	1.91	2.7491 (18)	177
C15—H15···O2^ii^	0.95	2.55	3.486 (2)	169
C8—H8A···O5^iii^	0.99	2.44	3.095 (2)	124

Symmetry codes: (i) −*x*+1, −*y*+1, −*z*+1; (ii) −*x*+1, −*y*, −*z*+1; (iii) −*x*+1, −*y*+1, −*z*+2.
